# Application of the Human Factors Analysis and Classification System (HFACS): Our Learning Journey and Challenges as a Psychiatric Institution

**DOI:** 10.1192/bjo.2026.11497

**Published:** 2026-06-30

**Authors:** Giles Ming-Yee TAN, Alias Lijo, Wei Xing, Asri Sapari, Audrey Yin-Chen Tan

**Affiliations:** Institute of Mental Health, Singapore, Singapore

## Abstract

**Aims:**

To evaluate the Institute of Mental Health's (IMH) clinical governance and quality processes following an independent review of systems related to care lapses, and to implement standardised root cause analysis (RCA) processes to improve patient safety outcomes.

**Methods:**

An independent review of IMH's clinical service delivery model and governance processes was conducted, examining Serious Reportable Events (SRE) and Sentinel Events (SE) from January 2017 to October 2023. A two-pronged approach was employed: an independent-led review by the National Healthcare Group and an internal self-review. Further in-depth analyses of selected incidents were conducted by subject matter experts using selection criteria based on recency, incident type, severity of harm, and findings. Root causes and recommendations were assessed using the New South Wales Root Cause AnalysisReview Committee's Classification of Recommendations. Following identification of governance deficiencies, a comprehensive change management process was implemented incorporating six key strategies: educate, engage, empower, evaluate, encourage, and escalate. A standardised RCA process utilising the Human Factors Analysis and Classification System (HFACS) framework was adopted, with establishment of the SRE-SE Oversight Panel in October 2024 to ensure oversight and consistent quality of the RCAs.

**Results:**

The review identified inadequately robust clinical and quality governance processes. Previous RCA investigations were sub-optimal, resulting in weak recommendations and reactive responses that missed opportunities for systems-level improvement. Prior to HFACS implementation, majority of recommendations in 2022 and 2023 were classified as 'weak', relying heavily on memory and vigilance. Post-implementation, there was an increase in robust, system-level recommendations requiring greater senior management involvement. However, approval processes initially extended to 373 days, failing to meet the 60-day requirement stipulated by the Ministry of Health. Following establishment of the SRE-SE Oversight Panel, approval times reduced from an average of 145 days to 50 days by April 2025. Despite improved recommendation quality, there was a decrease in the percentage of recommendations implemented within stipulated timeframes, attributed to the increased complexity of system-level interventions.

**Conclusion:**

The implementation of standardised RCA processes using HFACS successfully improved the quality and robustness of safety recommendations at IMH. However, stronger system-level recommendations require greater resources and extended implementation timelines. Continuous improvement through iterative Plan–Do–Study–Act cycles, stakeholder engagement, and appropriate escalation processes are essential for drivingsustainable change in psychiatric healthcare quality and safety initiatives.

